# Incretin-Related Pathology and Serum Exosome Detection in Experimental Alcohol-Related Brain Damage

**DOI:** 10.3390/biom15121670

**Published:** 2025-11-30

**Authors:** Suzanne M. de la Monte, Ming Tong, Yiwen Yang

**Affiliations:** 1 Departments of Pathology and Laboratory Medicine, Neurology, Neurosurgery and Medicine, Rhode Island Hospital, Alpert Medical School of Brown University, Brown University Health, Providence, RI 02903, USA; 2 Department of Medicine, Rhode Island Hospital, Alpert Medical School of Brown University, Brown University Health, Providence, RI 02903, USA; mtong216@gmail.com; 3 Biotechnology Graduate Program, Brown University, Providence, RI 02912, USA; yiwenyang@umass.edu

**Keywords:** alcohol, exosome, serum, brain pathology, incretin, amylin, insulin, multiplex ELISA, glucagon-like peptide-1, rat model

## Abstract

Alcohol’s chronic neurotoxic and degenerative effects mediate alcohol-related brain damage (ARBD), which is marked by neurobehavioral, cognitive, and motor deficits. Major underlying abnormalities include impairments in signaling through the insulin and insulin-like growth factor (IGF) pathways, which regulate energy metabolism. This study examined the potential role of dysregulated incretin network-related mechanisms as mediators of ARBD and evaluated a non-invasive serum exosome (S-EV)-based approach for detecting brain abnormalities. Frontal lobe tissue and S-EVs isolated from Long–Evans adolescent rats maintained for 2 weeks on control or 24% ethanol (caloric) containing liquid diets (n = 8/group) were analyzed using multiplex magnetic bead-based enzyme-linked immunosorbent assays (ELISAs). ARBD was associated with significantly reduced insulin, C-peptide, glucagon, ghrelin, leptin, GIP, and amylin levels in the frontal lobe and/or S-EV samples. In contrast, chronic ethanol exposure had no significant effects on PP, PYY, or GLP-1, and it did not increase proinflammatory cytokine expression. Chronic ethanol feeding broadly affected (primarily inhibiting) the expression of metabolic hormones linked to insulin/IGF signaling. The reductions in GIP and amylin suggest potential targets for therapeutic intervention to enhance brain energy metabolism via insulin networks. On the other hand, the findings suggest that GLP-1 receptor agonists may have limited efficacy in remediating the effects of ARBD. Finally, the results support the use of non-invasive S-EV assays to detect and guide treatment for metabolic brain dysfunction in ARBD.

## 1. Introduction

The neurotoxic and degenerative effects of chronic heavy alcohol consumption cause alcohol-related brain damage (ARBD) [1,2,3,4,5], in which cellular metabolic, homeostatic, and survival functions are broadly impaired [6]. Attendant cell loss and declines in neuronal conductivity and synaptic integrity [7,8,9,10] lead to deficits in cognition, behavior, and motor functions [2,11,12,13]. The underlying pathophysiology of ARBD is brain metabolic dysfunction linked to impaired insulin/insulin-like growth factor (IGF) signaling [14,15,16,17,18,19] at all levels within the cascade [15,20,21]. However, the most proximal/upstream abnormalities, including reduced insulin/IGF receptor binding and activation of receptor tyrosine kinases, compromise downstream pathways that mediate critical functions such as cell growth and survival, neuronal plasticity, myelin maintenance, and energy metabolism [14,16,17,22,23], mimicking the effects of insulin and IGF resistance. Moreover, ARBD can be associated with declines in trophic factors, further impairing signal transduction networks. To effectively improve ARBD-related dysregulation of metabolic networks, additional research is needed to understand the underlying causes. In this regard, the potential role of impaired integrin-related functions should be considered, given their broad roles in energy metabolism and insulin signaling [24,25].

The incretin family of hormones plays a critical role in maintaining metabolic homeostasis through interactions with receptors expressed in virtually all cell types. In the central nervous system (CNS), incretin mRNAs encoding glucagon-like peptide 1 (GLP-1) and glucose-dependent insulinotropic polypeptide (GIP) have been localized to the hypothalamus and other brain regions [26]. GLP-1, now the most studied of the incretins, stimulates glucose-dependent insulin secretion and insulin biosynthesis, promotes healthy insulin signaling, regulates blood glucose, inhibits glucagon secretion and gastric emptying, and curtails food intake. GLP-1 receptor agonists (RAs) and GIP-RAs are of particular interest because strong preclinical and clinical data show that, beyond their insulin-stimulating effects to achieve glycemic control, these drugs have pleiotropic CNS actions and they positively impact neuronal functions such as energy homeostasis, neurogenesis, and neuroprotection [27].

The mechanistic effects of incretins include reductions in neuroinflammation, oxidative stress, dysregulated metabolism, and impairments in plasticity and cell survival [27]. Given that GLP-1R is expressed on all CNS cell types (neurons, oligodendroglia, astrocytes, microglia, endothelial cells, pericytes) [28], and insulin/IGF signaling is critical for maintaining the integrity of brain structures targeted by the neurotoxic and degenerative effects of alcohol [14,29], it is of interest to explore the potential role of ethanol-impaired incretin signaling in relation to ARBD. Apart from their potential benefits related to the structural pathology of ARBD, clinical trials have been launched to address AUD-related neurobehavioral problems such as craving and addiction, which lead to ARBD [30,31,32,33,34]. The long-term outcomes of those investigations are still pending. Beyond incretins/incretin receptor agonists are the incretin-related molecules such as amylin, which is co-secreted with insulin and has neuroprotective and pro-metabolic effects in the brain, and oxyntomodulin, which acts as a dual agonist for GIP and GLP-1, and exerts neuroprotective, neurotrophic, and pro-metabolic effects in the brain [35]. Interest in these molecules has grown from the realization that targeting metabolic pathways to restore brain function will likely require multi-pronged approaches.

In addition to understanding mediators of ARBD, the need for more effective, timely treatment interventions underscores the importance of early detection and the capacity to monitor therapeutic effects. To accomplish these goals, it will be important to develop sensitive noninvasive assays. Extracellular vesicles (EVs), which are membranous nanoparticles that bear phenotypic features and molecular cargo characteristic of their cells of origin [36], traverse vascular networks and are detectable in biological fluids, including serum, saliva, cerebrospinal fluid, and extracellular tissue fluid. Many fields have begun to utilize EVs from body fluids to non-invasively diagnose and monitor diseases such as malignancies, neurodevelopmental defects, and neurodegeneration [37,38,39,40]. The bottleneck to achieving success lies in the limitations of accurately detecting and validating pathological changes using streamlined approaches suitable for clinical applications. Non-neoplastic metabolic CNS disorders are in dire need of novel tools to accelerate diagnoses and provide rapid, objective, quantifiable means of monitoring treatment responses. The recent finding that chronic ethanol exposure-mediated alterations in oligodendrocyte/white matter membrane-lipid glycoprotein and sphingolipid abnormalities can be detected in EVs isolated from white matter [41], inspired further investigations of clinically relevant liquid biopsy diagnostic approaches for ARBD. In this study, we utilized an established experimental model of ARBD to characterize incretin network-related pathology in the brain and determined the degree to which the brain abnormalities were detectable in EVs isolated from serum (S-EVs).

## 2. Materials and Methods

### 2.1. Experimental Model

Long Evans 4-week-old male and female adolescent rats (n = 8/group; 4 males and 4 females) (Charles River Laboratories, Wilmington, MA, USA) were pair-housed in same-sex cages in a pathogen-free animal facility with an automated 12 h light/dark cycle (Lights on at 7:00 AM and Lights off at 7:00 PM). Young adolescent rats were used in this experiment due to their heightened sensitivity to the neurotoxic effects of alcohol and, therefore, pronounced need for therapeutic intervention to mitigate against the long-term effects. After 7 days of acclimation, including 4 days of liquid diet adaptation, the rats were fed isocaloric Lieber-DiCarli liquid diets (BioServ, Frenchtown, NJ, USA) containing 0% (control) or 24% ethanol by caloric content for 2 weeks [42]. A two-week ethanol exposure period was used because, in a recent study, we demonstrated that the duration was sufficient to produce long-lasting alterations in brain metabolic signaling and white matter pathology [43]. The rats were pair-fed to ensure equivalent food intake in all groups. At the experimental endpoint, the rats were placed under deep isoflurane anesthesia. Fresh blood was obtained to measure alcohol and glucose concentrations, and hemoglobin-free serum separated from cardiac puncture blood was aliquoted and stored at −80 °C for subsequent S-EV isolation. The rats were not fasted prior to sacrifice since fasting could have confounded the results by causing alcohol-withdrawal states in the ethanol-fed groups. Following euthanasia, the frontal lobes were dissected and stored fresh at −80 °C. The use of rats in these experiments was done in compliance with the National Institutes of Health (NIH) Guide for the Care and Use of Laboratory Animals and approved by the Institutional Animal Care and Use Committee (IACUC) at Rhode Island Hospital and Brown University Health (Committee #504020 approved 16 September 2023).

### 2.2. Tissue Homogenization for Immunoassays

Frontal lobe tissue used in this experiment was obtained by making a 3 mm thick pre-temporal coronal slice section, excluding the leptomeninges, cerebral vasculatures, olfactory bulbs and olfactory tracts. The fresh tissue was rapidly frozen on dry ice with the plane of section and orientation preserved. Immediately prior to use, ~50 mg aliquots of cortex with underlying white matter each were homogenized in 5-volumes of weak lysis buffer (50 mM Tris (pH 7.5), 150 mM NaCl, 5 mM EDTA (pH 8.0), 50 mM NaF, 0.1% Triton X-100) supplemented with protease (1 mM PMSF, 0.1 mM TPCK, 2 µg/mL pepstatin A, 2 µg/mL aprotinin, 1 µg/mL leupeptin) and phosphatase (10 mM Na_3_VO_4_) inhibitors. Stainless steel beads (5 mm diameter) were added to each sample for homogenization in a TissueLyser II instrument (Qiagen, Germantown, MD, USA). Centrifuge-clarified (14,000× *g* for 15 min at 4 °C) supernatants were aliquoted and stored at −80 °C. Protein concentrations were measured with the bicinchoninic acid (BCA) assay. Frontal lobe tissue was selected for study because it is a major target of ARBD.

### 2.3. Serum Exosome Isolation

Exosomes were isolated from serum using the Total Exosome Isolation reagents (Invitrogen/Life Technologies, Carlsbad, CA, USA) following the manufacturer’s protocol. In brief, frozen serum from non-hemolyzed samples was thawed in a 25 °C water bath and then centrifuged at 2000× *g* for 30 min to remove debris. A 100 µL aliquot of clarified serum was added to 20 µL of total exosome isolation reagent, vortexed to achieve a homogeneous solution, incubated at 4 °C for 30 min, and then centrifuged at 10,000× *g* for 10 min at room temperature. The pelleted exosomes were resuspended in 25 µL phosphate-buffered saline (PBS). After removing an aliquot for nanoparticle tracking analysis, the remaining sample was homogenized in weak lysis buffer. Protein concentration was measured with the BCA assay.

### 2.4. Nanoparticle Tracking Analysis (NTA)

NTA was performed with a NanoSight NS500 instrument (Malvern Instruments, Malvern, UK) equipped with a syringe pump, and NTA software NTA 3.0 (MilliporeSigma, Burlington, MA, USA) to determine the size distributions and number of particles per sample. For the NTA studies, DMSO was added to a final concentration of 1% for stabilization. The samples were analyzed in triplicate. Prior to sample analysis, the instrument was calibrated using Nanosphere Standard Beads (Thermo Scientific, Waltham, MA, USA). Subsequent continuous monitoring ensured the maintenance of optimized settings. Video recordings were used to evaluate the mean, median, and mode of particle size and concentration.

### 2.5. Multiplex Enzyme-Linked Immunosorbent Assays (ELISAs)

A rat 13-plex metabolic magnetic bead-based panel (Millipore RMHMAG-84K; Burlington, MA, USA) was used to measure amylin, C-peptide, Ghrelin, Leptin, Glucagon, glucose-dependent insulinotropic polypeptide (GIP), glucagon-like peptide 1 (GLP-1), pancreatic polypeptide (PP), Peptide YY/Neuropeptide Y (PYY), insulin, Interleukin 6 (IL-6), Monocyte chemoattractant protein-1 (MCP-1), and tumor necrosis factor-alpha (TNF-α) in frontal lobe (FL) tissue and serum exosomes (See Supplementary Table S1 for molecule functions). The assays were performed according to the manufacturer’s protocols. In brief, after incubating 200 µg protein samples with antibody-conjugated magnetic beads, immunoreactivity was detected with biotinylated secondary antibodies and Streptavidin-conjugated phycoerythrin. Immunoreactivity was measured in a Milliplex MagPix Instrument, and the results were analyzed using the xPONENT software v4.3 (ThermoFisher, Plainville, MA, USA). Standard curves for each analyte were included in all assays.

### 2.6. Duplex ELISA

Exosome immunoreactivity to the CD9, CD63, and CD81 tetraspanins was measured by duplex ELISA with results normalized to large acidic ribosomal protein (RPLPO) as the loading control [41,44]. RPLPO immunoreactivity increases linearly with protein content between 10 and 80 ng/well [41]. To perform the duplex ELISAs, triplicate 50 ng protein samples, each in 50 µL bicarbonate binding buffer, were robotically distributed (EpMotion 5070, Eppendorf, Framingham, MA, USA) into 96-well MaxiSorp plates. After overnight adsorption at 4 °C, non-specific binding sites were masked with Superblock TBS. Then, the samples were incubated with primary antibodies (0.2–5.0 µg/mL) overnight at 4 °C. Immunoreactivity was detected with horseradish peroxidase (HRP)-conjugated secondary antibodies and the Amplex UltraRed soluble fluorophore. Fluorescence intensity was measured (Ex 530 nm/Em 590 nm) in a Spectra-Max M5 Multimode Plate Reader (Molecular Devices, Sunnyvale, CA, USA). After rinsing in Tris-buffered saline (TBS), the samples were incubated with biotin-conjugated anti-RPLPO, followed by streptavidin-conjugated alkaline phosphatase, and RPLPO immunoreactivity was detected with 4-Methylumbelliferyl phosphate (4-MUP) (Ex 360 nm/Em 450 nm). Fluorescence was measured in a SpectraMax M5. The calculated ratios of target protein to RPLPO were used for statistical comparisons.

### 2.7. Data Analysis

GraphPad Prism 10.4 software (GraphPad Software Inc., Boston, MA, USA) was used to analyze data and generate graphs. The GraphPad data analysis tools employed automatically evaluated the data to ensure the assumptions for normality were met. Violin plots depict the distribution of results with the median (mid-horizontal bar), the first (lower horizontal line) and third (upper horizontal line) quartiles, and range (tips). Inter-group differences in evaluating effects of ethanol exposure and duration of ethanol exposure were compared by Welch *t*-test analysis with correction for multiple comparisons. Software-generated statistically significant (*p* ≤ 0.05) differences are displayed within the graph panels. The data were analyzed using a false discovery rate of 0.10.

## 3. Results

### 3.1. Rat Model Characteristics

Initial analysis of the data failed to detect significant effects of sex, corresponding with results obtained with the same model and longer-term ethanol exposures [43]. Therefore, the results obtained from male and female rats were combined and analyzed together. Table 1 summarizes the model characteristics. The control and ethanol-fed groups each included 4 males and 4 females. Significantly higher mean blood alcohol concentrations were measured in the alcohol diet group (*p* < 0.0001). In contrast, there were no significant differences in body weight, brain weight, or blood glucose concentration.

### 3.2. S-EV Characteristics

**Figure 1:**
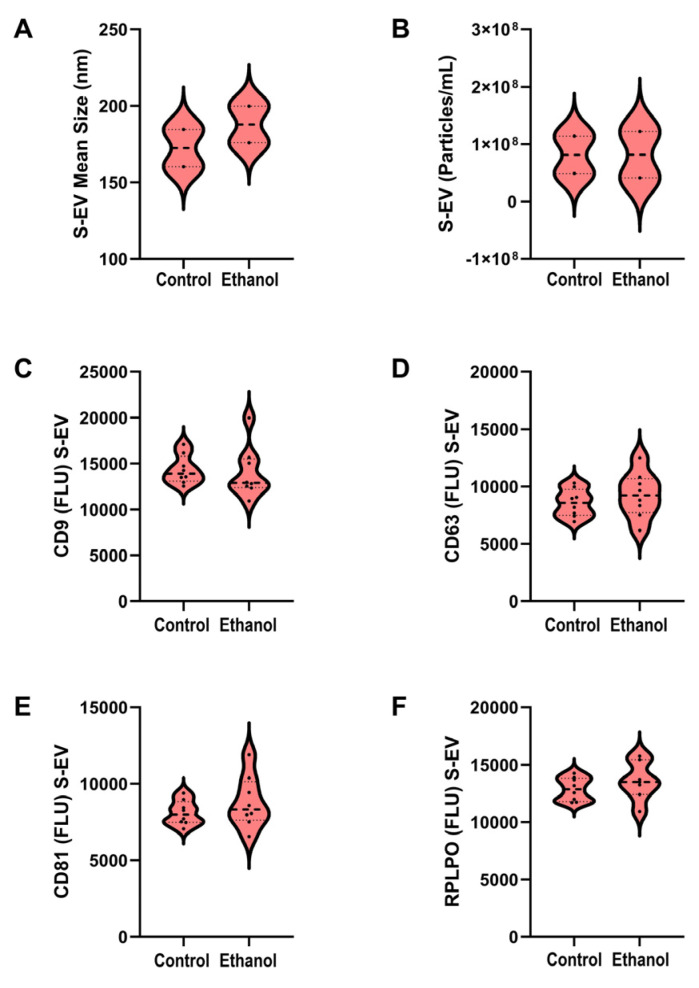
Serum extracellular vesicle (S-EV) characteristics. S-EVs were isolated from control and chronic ethanol-fed Long Evans rats (n = 8/group). NanoSight Tracking Analysis (NTA) demonstrated similar nanoparticle (**A**) mean diameters and (**B**) concentrations in the control and ethanol group S-EV samples. Duplex ELISAs measured tetraspanin (**C**) CD9, (**D**) CD63, and (**E**) CD81, and (**F**) RPLPO immunoreactivity in 50 ng S-EV protein samples. Inter-group comparisons made by Welch T-test detected no significant (*p* ≤ 0.05) differences for any of the measurements.

Nanosite tracking analysis demonstrated similar EV mean diameters (Figure 1A) and particle concentrations (Figure 1B) in control and ethanol-exposed samples. Duplex ELISAs used to measure tetraspanin immunoreactivity revealed similar levels of CD9 (Figure 1C), CD63 (Figure 1D), and CD81 (Figure 1E) in the control and ethanol S-EV samples. In addition, HSP70 immunoreactivity was similar for the two groups (Figure 1F).

### 3.3. Pro-Inflammatory Cytokines

**Figure 2:**
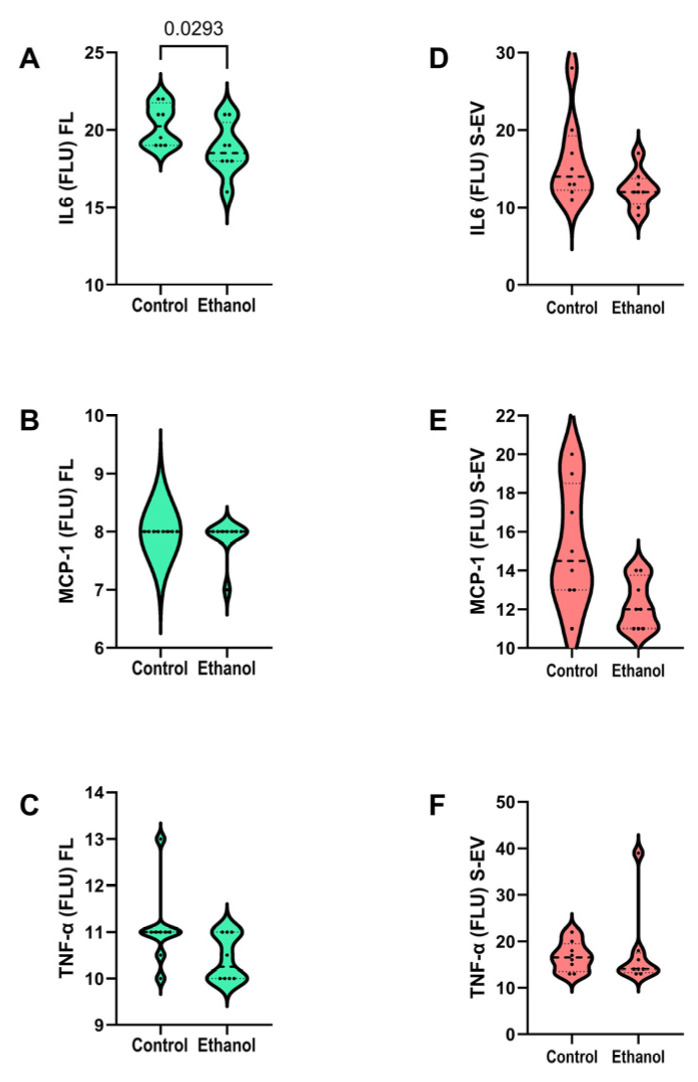
Effects of chronic ethanol feeding on proinflammatory cytokine expression in frontal lobe tissue and serum extracellular vesicles (S-EVs). Long Evans rats were fed with isocaloric liquid diets containing 0% (control) or 24% caloric ethanol for 2 weeks after a period of adaptation (n = 8/group). Frontal lobe (**A**–**C**) and S-EV (**D**–**F**) levels of (**A**,**D**) Interleukin 6 (IL6), (**B**,**E**) MCP-1, and (**C**,**F**) Tumor necrosis factor-α (TNF-α) were measured by multiplex ELISA. Immunoreactivity is expressed as fluorescent light units. Inter-group differences were compared with Welch *t*-tests. Significant results (*p* ≤ 0.05) are displayed over the graphs.

The frontal lobe level of the IL6 pro-inflammatory cytokine (Figure 2A) was significantly reduced in the ethanol-exposed group, whereas the levels of MCP-1 (Figure 2B) and TNF-α (Figure 2C) were similar in the control and ethanol frontal lobe samples. In contrast to the findings in frontal lobe tissue, the S-EV levels of IL6 (Figure 2D), MCP-1 (Figure 2E), and TNF-α (Figure 2F) were similarly abundant in the control and ethanol-exposed groups.

### 3.4. Insulin, C-Peptide, and Glucagon

**Figure 3:**
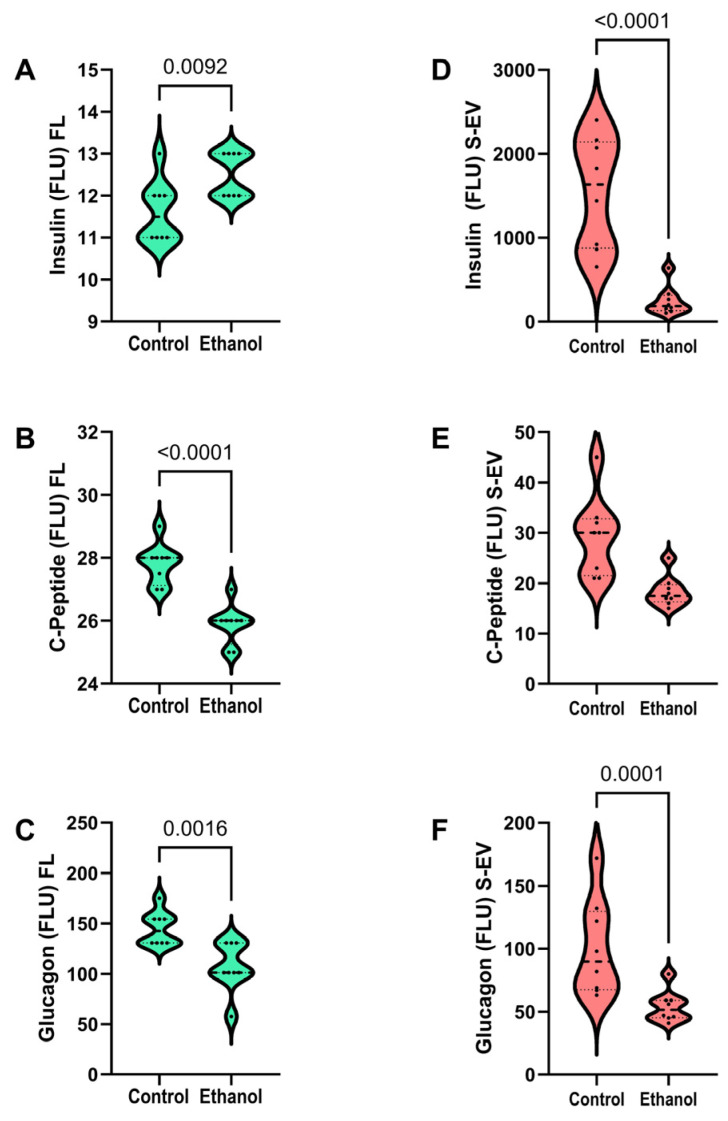
Effects of chronic ethanol feeding on frontal lobe and S-EV expression of insulin-related hormones. A multiplex magnetic bead-based ELISA was used to measure immunoreactivity (fluorescent light units; FLU) to (**A**,**D**) Insulin, (**B**,**E**) C-Peptide, and (**C**,**F**) glucagon in frontal lobe tissue homogenates (**A**–**C**) and S-EVs (**D**–**F**) of Long Evans rats that were fed for 2 weeks with isocaloric liquid diets containing 0% (control) or 24% caloric ethanol (n = 8/group). Inter-group differences were compared with Welch *t*-tests. Significant results (*p* ≤ 0.05) are displayed in the graph panels.

Analysis of the insulin, C-peptide, and glucagon cluster revealed significantly higher levels of insulin (Figure 3A), and lower levels of C-peptide (Figure 3B) and glucagon (Figure 3C) in the ethanol-exposed relative to control frontal lobe samples. Regarding the S-EV analyses, significant ethanol-associated reductions in insulin (Figure 3D) and glucagon (Figure 3F) were observed. Although S-EV C-Peptide was also somewhat reduced by chronic ethanol feeding (Figure 3E), the inter-group differences did not reach statistical significance.

### 3.5. Ghrelin, Leptin, and Pancreatic Polypeptides

**Figure 4:**
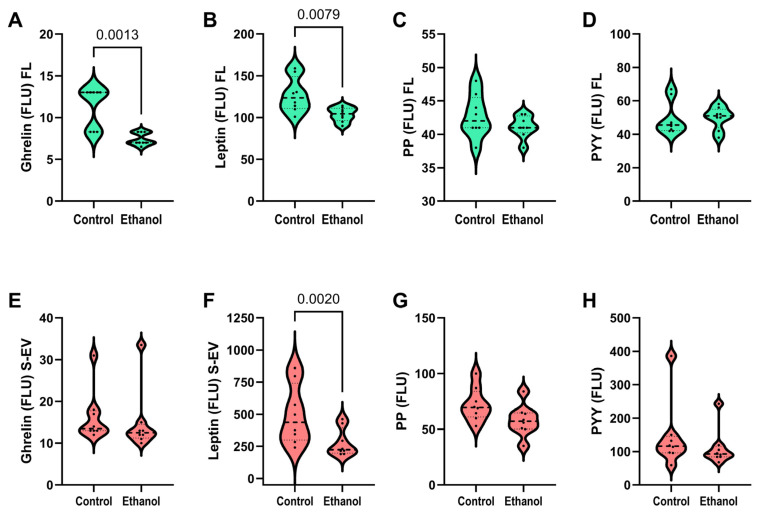
Impact of chronic ethanol exposure on gut hormone polypeptide expression. including incretins. Frontal lobe tissue homogenates and S-EVs were prepared from Long Evans rats fed for 2 weeks with isocaloric liquid diets containing 0% (control) or 24% caloric ethanol (n = 8/group). A multiplex magnetic bead-based ELISA was used to measure frontal lobe (**A**–**D**) and S-EV (**E**–**H**) immunoreactivity (fluorescent light units; FLU) to (**A**,**E**) ghrelin, (**B**,**F**) leptin, (**C**,**G**) PP, and (**D**,**H**) PYY. Immunoreactivity was quantified using standards for calculating protein concentration. Inter-group differences were compared with Welch *t*-tests. Significant results (*p* ≤ 0.05) are displayed in the graph panels.

Analysis of the ghrelin, leptin, PP, and PYY cluster revealed significant ethanol-associated reductions in frontal lobe levels of both ghrelin (Figure 4A) and Leptin (Figure 4B) immunoreactivity. For the S-EV samples, ethanol also significantly reduced leptin immunoreactivity (Figure 4F), but, discordant to the frontal lobe, it had no significant effect on ghrelin (Figure 4E). There were no significant effects of chronic ethanol feeding on PP or PYY immunoreactivities in frontal lobe tissue (Figure 4C,D) and S-EVs (Figure 4G,H).

### 3.6. Incretin Network Cluster

**Figure 5:**
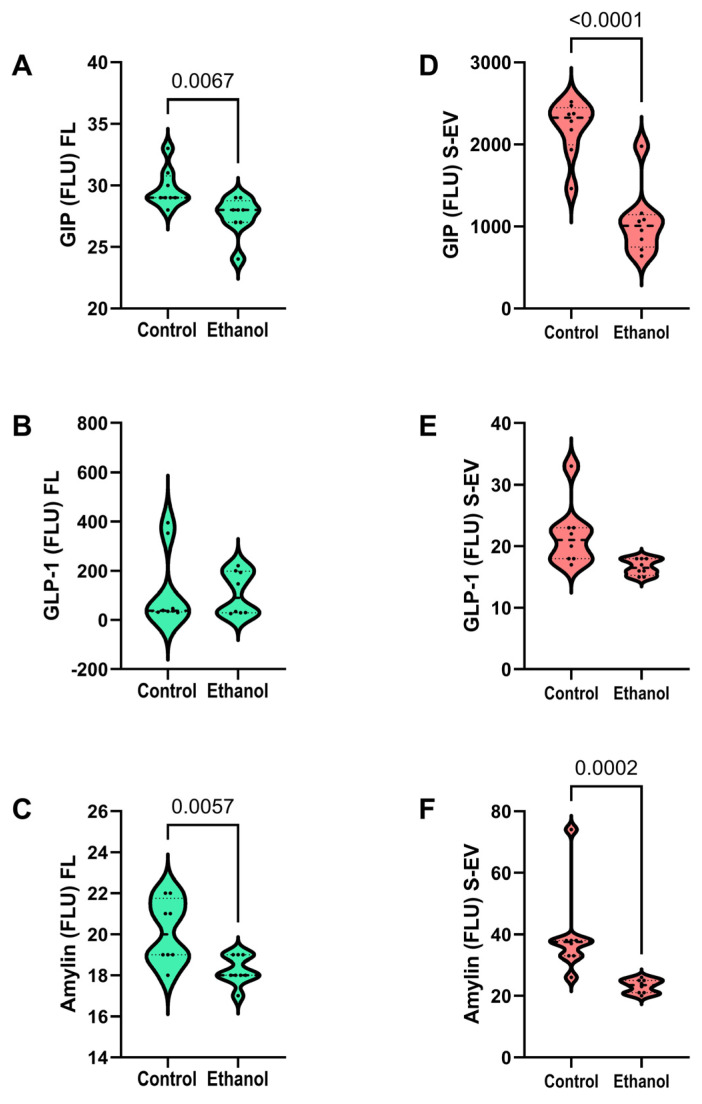
Effects of chronic ethanol feeding on frontal lobe and S-EV expression of incretins. Long Evans rats were fed with isocaloric liquid diets containing 0% (control) or 24% caloric ethanol for 2 weeks after a period of adaptation (n = 8/group). Frontal lobe (**A**–**C**) and S-EV (**D**–**F**) levels of (**A**,**D**) GIP, (**B**,**E**) GLP-1, and (**C**,**F**) amylin were measured using a magnetic bead-based multiplex ELISA panel. Results (fluorescent light units, FLU) were calculated based on internal standards. Inter-group differences were compared with Welch *t*-tests. Significant results (*p* ≤ 0.05) are displayed in the graph panels.

Analysis of GIP, GLP-1, and amylin immunoreactivities revealed significant ethanol-mediated reductions in GIP (Figure 5A,D) and amylin (Figure 5C,F) in both frontal lobe and S-EV samples. In contrast, chronic ethanol feeding had no significant effects on GLP-1 expression in either the frontal lobe (Figure 5B) or S-EVs (Figure 5E). The concordant ethanol-associated declines in frontal lobe and S-EV GIP and amylin immunoreactivities represent a novel finding of potential diagnostic and therapeutic targeting importance.

## 4. Discussion

In ARBD, impaired insulin/insulin-like growth factor (IGF) signaling is a crucial underlying pathophysiological mediator of neurodegeneration [14,15,16,17,18,19]. Disruption of the insulin/IGF signaling network at multiple levels within the cascade compromises energy metabolism and homeostasis, which are needed for cell survival, neuronal plasticity, and white matter integrity [14,16,17,22,23]. Although it is well known that short-term ethanol exposures inhibit phosphorylation of molecules that drive intracellular signaling, the underlying mediators of ethanol’s long-term adverse effects are not fully understood. Growing appreciation of the broad critical roles of incretin signaling in maintaining metabolic homeostasis and insulin network function throughout the body led to the hypothesis that the chronic adverse effects of ethanol may be mediated by the inhibition of integrin-related networks.

The incretin family of hormones interacts with receptors throughout the body, including the CNS. GLP-1 stimulates glucose-dependent insulin secretion and insulin biosynthesis, promotes healthy insulin signaling, regulates blood glucose, inhibits glucagon secretion and gastric emptying, and curtails food intake. GIP stimulates insulin secretion and lipoprotein lipase, maintains glucose homeostasis, and modulates fatty acid metabolism. Beyond their insulin-stimulating effects for glycemic control, GLP-1 and GIP receptor agonists exert pleiotropic CNS actions that impact neuronal energy homeostasis, neurogenesis, and neuroprotection [27]. Incretins confer neuroprotection by reducing neuroinflammation, oxidative stress, metabolic dysregulation, impairments in plasticity, and cell death [27]. Beyond incretins/incretin receptor agonists are the incretin-related molecules such as amylin and oxyntomodulin. Amylin is co-secreted with insulin and has neuroprotective and pro-metabolic effects in the brain. Oxyntomodulin acts as a dual agonist for GIP and GLP-1, and exerts neuroprotective, neurotrophic, and pro-metabolic effects in the brain [35]. Interest in these molecules has grown as it has become clear that targeting metabolic pathways to restore brain function will likely require multi-pronged approaches. Studies to characterize the role of ethanol-impaired incretin signaling in relation to ARBD are significant because GLP-1R is expressed on all CNS cell types (neurons, oligodendroglia, astrocytes, microglia, endothelial cells, and pericytes) [28]. In addition, insulin/IGF signaling is critical for maintaining CNS structures and functions that are targeted in ARBD. Finally, therapeutic measures to restore incretin signaling networks potentially could be re-purposed to treat ARBD.

This study characterizes the effects of chronic ethanol feeding on the expression of insulin- and incretin-network molecules in the brain. The study had two main objectives. The first was to better delineate the features of metabolic dysregulation associated with known ARBD-related impairments in insulin/IGF signaling. The second goal was to determine the potential feasibility of utilizing a serum EV approach to non-invasively detect ARBD-related brain metabolic abnormalities. The research was based on a well-characterized experimental model of ARBD in which cognitive and motor impairments, brain abnormalities in insulin/IGF signaling through the insulin/IGF-1 receptors, IRS, and Akt, and alterations in oligodendrocyte/myelin mRNA, protein, and lipid expression have been documented [6]. Of note is that a potential limitation of the results interpretation is that, although the samples were heavily enriched for exosomes using commercial precipitation methods, soluble molecules not directly bound to exosomes could potentially be included in the analyses [45].

Regarding inflammatory cytokines, ethanol feeding did not activate pro-inflammatory cascades mediated by IL6, MCP-1, or TNF-α in either frontal lobe brain tissue or S-EVs. Instead, one cytokine, IL6 was reduced in frontal lobe tissue by ethanol exposure, indicating that increased cytokine-mediated neuroinflammatory responses were not detected as features of this model, consistent with previous observations [46]. These findings are corroborated by the absence of pro-inflammatory responses in the S-EVs from chronic ethanol-fed rats. Instead, given the increasingly recognized additional positive roles cytokines play in neurogenesis, the ethanol-associated down-regulation of brain pro-inflammatory cytokines could reflect an ethanol-mediated decline in synaptic plasticity [47].

Chronic ethanol exposure increased frontal lobe but reduced S-EV insulin, whereas for C-Peptide, which is a more stable marker of insulin expression, the levels were concordantly reduced in the brain and not significantly altered in S-EVs. It is noteworthy that insulin immunoreactivity was approximately 100-fold higher in the S-EVs than in brain tissue, most likely due to dominant peripheral/systemic sources of insulin. The significantly reduced insulin immunoreactivity in ethanol-exposed S-EVs suggests that chronic ethanol exposure broadly inhibits insulin expression/secretion, which likely contributes to systemic metabolic dysregulation. Although the higher levels of frontal lobe insulin in the ethanol group could reflect increased uptake from the periphery in response to reduced C-peptide synthesis, an alternative explanation is that pro-insulin processing is impaired, or EV cargo selection is dysregulated by ethanol exposure. In contrast, the similar levels of C-Peptide expression in frontal lobe tissue and S-EVs support the concept that its synthesis and regulation occur both in the CNS and periphery. The findings suggest that chronic ethanol feeding reduces endogenous brain insulin availability, compromising metabolic function. Furthermore, the ethanol-associated reductions in both frontal lobe and S-EV glucagon imply that these abnormalities also have important roles in the dysregulation of glucose homeostasis and metabolism in both ARBD and systemic alcohol-related pathology.

Frontal: lobe tissue from chronic ethanol-exposed rats had reduced levels of leptin and ghrelin immunoreactivity, corresponding with previous observations in human subjects following ethanol consumption [46] or exposure [48], and in people with Alzheimer’s disease neurodegeneration [49]. Given the opposing roles of these molecules in relation to energy expenditure and intake, their simultaneously reduced brain levels could be linked to their alternative functions. Beyond regulating food intake and body weight, ghrelin and leptin have important roles in synaptic plasticity utilized in response to changes in energy status and metabolism [50,51]. Therefore, their reductions in ARBD could reflect declines in synaptic plasticity. The delicate balance of restoring these functions while dampening craving is an important therapeutic consideration since a further reduction in ghrelin and leptin could adversely impact cognitive behavior and brain metabolic function. It is noteworthy that S-EV leptin levels were also reduced by ethanol, whereas S-EV ghrelin was unaffected. Whether ethanol’s inhibitory effects on ghrelin in S-EVs reflected responses in the brain or peripheral tissue insulin resistance associated with chronic ethanol feeding requires further investigation. Chronic ethanol exposure effects on PP or PYY immunoreactivity were not observed in either frontal lobe tissue or S-EVs, suggesting that in this model, chronic ethanol exposure did not adversely impact the neuropeptide Y family of peptides or their signaling networks.

Most interesting was the finding of concordant significant reductions in GIP and amylin, vis-à-vis unaffected GLP-1 in frontal lobe tissue and S-EVs from the chronic ethanol-fed rats. The fact that chronic ethanol exposure did not significantly impact GLP-1 in either the frontal lobe or S-EVs argues against that component of integrin-network signaling being disrupted in ARBD. However, a note of caution is needed before suggesting that therapeutic interventions with agonists that target GLP-1 receptors may not be beneficial in ARBD because longer term exposures could potentially have different, perhaps more damaging effects on GLP-1. On the other hand, the finding of significantly reduced GIP and amylin, which have important regulatory roles in insulin secretion, glucose homeostasis, and fatty acid metabolism, is important. The clinical/translational relevance is that their corresponding receptors may be exceptional targets for restoring the insulin network and metabolic functions in ARBD. Furthermore, the incretin-related molecule, amylin, is co-secreted with insulin and has neuroprotective and pro-metabolic effects in the brain. In addition, oxyntomodulin, another incretin-related molecule not investigated herein, acts as a dual agonist for GIP and GLP-1, exerts neuroprotective, neurotrophic, and pro-metabolic effects in the brain [34]. Interest in these molecules has grown due to the realization that targeting metabolic pathways to restore brain function will likely require multi-pronged approaches. Therefore, it will be important to consider the potential use of receptor agonists for amylin, oxyntomodulin, and other compounds capable of supporting insulin/IGF metabolic functions. This concept is feasible because extant pharmacological compounds could be repurposed to treat ARBD-associated impairments in insulin signaling. Clinical trials to address alcohol use disorder behaviors that lead to ARBD [30,31,32,33,34,35,36] have been launched, but their long-term outcomes are still pending. Although these types of therapeutic agents are still in clinical trials [52,53,54], preclinical studies indicate their further relevance to brain metabolic dysfunction [24], including in association with ARBD and Alzheimer’s disease [19,21].

## 5. Conclusions

Among the 10 peptide hormones tested, amylin, GIP, glucagon, and leptin had concordant reductions in immunoreactivity in both frontal lobe tissue and S-EVs from chronic ethanol-fed rats. This suggests that metabolic abnormalities linked to insulin signaling networks in the brain can be detected non-invasively in the peripheral blood via analysis of S-EVs. Importantly, these observations provide new evidence that sensitive liquid biopsy-type assays could be used to detect and monitor metabolic brain diseases caused by chronic alcohol exposure. Presumably, treatments that abrogate these adverse effects of ethanol would also be detectable in S-EVs. The results herein were obtained with unselected S-EVs that could have originated from various organs and tissues since all cell types produce EVs which readily circulate and traffic to various sites throughout the body. Future studies will serve to refine these analyses by characterizing the expression of insulin and incretin network metabolism-related molecules in S-EVs enriched for neuronal and glial (PDGFRα+ and O4+) subsets using magnetic bead separation technology. 

## Figures and Tables

**Table 1:** Long Evans Rat Ethanol Exposure Models.

2-Week Model	Control	Ethanol	*t*-Test (*p*-Value)
# Rats (Male/Female)	8 (4/4)	8 (4/4)	
Body Weight (g)	182.9 ± 22.72	170.6 ± 17.13	N.S.
Blood Alcohol (mg/dL)	25.76 ± 4.5	108.4 ± 11.2	<0.0001
Blood Glucose (mg/dL)	194.6 ± 18.17	192.4 ± 28.99	N.S.
Brain Weight (g)	1.708 ± 0.08	1.702 ± 0.05	N.S.

Intergroup comparisons of mean ± S.D. of body weight, blood alcohol concentration, blood glucose, brain weight and body were compared using the Welch *t*-test. Results were analyzed with Graphpad Prism 10.4. Calculated significant (*p* ≤ 0.05).

## Data Availability

The data underlying this article will be shared upon reasonable request to the corresponding author.

## Supplementary Materials

The following supporting information can be downloaded at https://www.mdpi.com/article/10.3390/biom15121670/s1, Table S1: Gut/Metabolic Hormone Panel.

## Abbreviations

The following abbreviations are used in this manuscript:

GIP	Glucose-dependent insulinotropic polypeptide
GLP-1	Glucagon-like peptide 1
PP	Pancreatic polypeptide
PYY	Peptide YY/Neuropeptide Y
IL-6	Interleukin 6
MCP-1	Monocyte chemoattractant protein-1
TNF-α	Tumor necrosis factor-alpha
CD9	Tetraspanin-29
CD63	Tetraspanin-30
CD81	Tetraspanin-28
HSP70	Heat shock protein 70
