# Supplementary material for: Incretin-Related Pathology and Serum Exosome Detection in Experimental Alcohol-Related Brain Damage
Source: Biomolecules. 2025 Nov 30;15(12):1670. doi: 10.3390/biom15121670 (PMC12731142; doi:10.3390/biom15121670)
Supplement: Supplementary file 1 [file biomolecules-15-01670-s001.zip › biomolecules-3995588-supplementary.pdf]

Supplementary Materials

Table S1. Gut/Metabolic Hormone Panel.

Factor	Gene Name	Functions
**Amylin**	*IAPP*; Islet amyloid polypeptide	Co-stored and co-secreted with insulin; controls blood glucose by inhibiting food intake and slowing gastric emptying; deficiency is associated with cognitive deficits and insulin deficiency
**C-Peptide**	*INS*; encoded by the insulin gene	Connecting peptide and stable by-product of pro-insulin cleavage to generate insulin; mediates efficient assembly, folding, and processing of insulin in the ER.
**Ghrelin**	*GHRL*; Ghrelin and Obestatin Prepropeptide	Ligand for growth hormone secretagogue receptor type 1; induces growth hormone release from the pituitary; regulates growth; stimulates appetite; induces adiposity; stimulates gastric acid secretion. Regulates synaptic function and plasticity related to feeding behavior, as well as cognition
**GIP**	*GIP* Glucose-dependent insulinotropic polypeptide; Gastric Inhibitory Polypeptide	Incretin: Potent stimulator of insulin secretion, maintains glucose homeostasis; stimulates lipoprotein lipase; modulates fatty acid metabolism; poor inhibitor of gastric acid secretion
**GLP-1**	*GCG;* Glucagon-like peptide 1	Incretin: Potent stimulator of glucose-dependent insulin release; stimulates glucose disposal, independent insulin actions; suppresses plasma glucagon; modulates gastric motility; may suppress satiety; promotes growth of intestinal epithelium; neuroprotective.
**Glucagon**	*GCG*	Regulates glucose metabolism and homeostasis by increasing gluconeogenesis and decreasing glycolysis and counter-regulatory to insulin; raises plasma glucose in response to insulin-induced hypoglycemia; initiates and maintains hyperglycemic conditions in diabetes mellitus.
**Insulin**	*INS*	Reduces blood glucose; regulates carbohydrate and lipid metabolism by increasing cell permeability to monosaccharides, amino acids, and fatty acids; accelerates the pentose phosphate cycle and glycogen synthesis in the liver
**Leptin**	*LEP*	Important regulator of energy balance by inhibiting food intake and promoting energy expenditure; helps regulate fat depots. Binds to leptin receptors in the brain to inhibit feeding and promote energy expenditure.
**PP**	*NPY*/Pancreatic Polypeptide	Neuropeptide Y family member peptide; In the brain, PP is NPY, which is abundant in the brainstem, thalamus, hypothalamus, subcortical nuclei, and cerebral cortex.
**PYY**	*Peptide YY*	Member of the neuropeptide Y (NPY) family of peptides; signals the brain to attenuate food intake, anxiety, and depression-related behavior; Gut-brain axis; postprandial secretion targets the cortex and hypothalamus
